# Analysis of a Salivary Calculus from a Horse

**Published:** 1858-01

**Authors:** A. Snowden Piggot


### ARTICLE II.

### *Analysis of a Salivary Calculus from a Horse.* By A. SNOWDEN PIGGOT, M. D.

THE subject of salivary calculus* has attracted no little attention from dentists and chemists, but owing to the comparative rarity of its occurrence, but few reliable facts, as to its chemical constitution, have been obtained. I have thought, therefore, that the following brief account of the physical characters and chemical constitution of a large calculus taken from the duct of Steno, in a horse, might be of interest.

The specimen was of a flattened cylindrical form, having a rounded blunt point at one end, and a rough, shallow concavity at the other. Its surface was generally smooth, excepting towards the base, or concave extremity, where it was rough and corrugated. The color, externally, was a

*I wish it to be understood that I use the term *salivary calculus,* to denote a hard body deposited within the ducts of the salivary glands, and not as it is often used by dentists, to signify the incrustation on the teeth, commonly called *tartar.*

pale fawn, but when the outer layer was removed it was brilliantly white. Its length was 2.88 inches, its thickness across the largest diameter, 1 inch, and across the shortest, 0.75 of an inch. It weighed 30.173 grammes, (465.691 grains, or nearly an ounce.) Its specific gravity, at the maximum density of water, was 2.207. It could be cut, or rather scraped, with some difficulty, by the knife, the outer portion being the hardest.

The quantity taken for analysis was smaller than I could have wished, because, as it was presented to the museum of the Baltimore College of Dental Surgery, I did not wish to disfigure so interesting a specimen. The calculus was, therefore, drilled entirely through, longitudinally, and the powder thus obtained mingled with what was procured by scraping the surface. It was discovered that the calculus was made up of concentric layers, gradually deposited around a nucleus, which was found to be a splinter of pine wood. This had probably entered the duct through a wound in the buccal surface of the cheek, and by its irritating action induced the deposit of calcareous matter from the salivary liquid.

The following table expresses the result of my analysis:

| | |
|---|---|
| Water, | 1.262 |
| Organic matter, | 8.350 |
| Lime, | 49.572 |
| Magnesia, | 2.820 |
| Alkalies, | 0.816 |
| Carbonic acid, | 31.702 |
| Phosphoric acid, | 2.320 |
| Silica, | 0.330 |
| Chlorine, | 0.747 |
| Sulphuric acid, | 2.019 |
| | 99.938 |

In attempting to construct a rational formula for this substance, it will be found that there is an excess of the alkaline earths, lime and magnesia. This is probably combined with some organic acid confounded under the generic term, organic matter. It would have been more satisfactory to have attempted the direct determination of this question, but for reasons already assigned, a sufficient amount for such an investigation could not be procured. It is abundantly evident, however, that the great bulk of the calculus (say 75 per cent.) is composed of the carbonate of lime; its other constituents being comparatively unimportant.

This analysis, therefore, corroborates those previously made of the salivary calculi of herbivora. There is a marked contrast between them and the calculi taken from the human subject. Although the analyses have been evidently made without much care, they reveal one fact, viz. that in the herbivora they are largely composed of carbonate of lime.

Thus Caventou obtained from one of these bodies taken from an ass:

| | |
|---|---|
| Carbonate of lime, | 91.6 |
| Phosphate of lime, | 4.8 |
| Animal matter soluble in water, | 3.6 |
| | 100.0 |

From one taken from a horse, Lassaigne obtained,

| | |
|---|---|
| Carbonate of lime, | 84 |
| Phosphate of lime, | 3 |
| Animal matter soluble in water, | 9 |
| Water, | 3 |
| | 99 |

Henry's results for a similar concretion from a horse were,

| | |
|---|---|
| Carbonate of lime, | 85.52 |
| Carbonate of magnesia, | 7.56 |
| Phosphate of lime, | 4.40 |
| Animal matter soluble in water, | 2.42 |
| | 99.90 |

I got less carbonate than any of these experimenters. The calcareous and magnesian salts, I make to be,

| | |
|---|---|
| Carbonate of lime, | 72.050 |
| Sulphate do. | 3.422 |
| Phosphate do. | 3.813 |
| Phosphate of magnesia, | 2.921 |

The rest of the lime and magnesia appear to be combined in some other form, probably, as I have already said, with organic matter or organic acids.

In man, on the other hand, much more phosphate of lime has been found. Poggiale obtained 94 per cent. of this salt in a human calculus.

Von Bibra, of Erlangen, met with eleven of these concretions in one man, a peasant, twenty-two years of age. He examined one of the lighter calculi, and found it to consist of a nucleus of albumen and mucus, round which the rest of the calculus had been gradually deposited in concentric layers. His analyses yielded him :

| | |
|---|---|
| Phosphate of lime, . . . . . | 38.2 |
| Carbonate of lime, . . . . . | 13.3 |
| Phosphate of magnesia, . . . . | 5.1 |
| Fatty matter with traces of soda, . . . | 3.1 |
| Organic matter, . . . . . . | 35.0 |
| Water, . . . . . . . . . | 5.3 |
| | 100.0 |

The reader will be struck with the difference between the amount of phosphates in human calculi, and in those from the herbivora. The same difference exists in the urine of these different classes of animals. Thus, in man, and still more in the carnivoras, this secretion abounds in phosphates, while in the herbivora carbonates predominate.

When we consider that the salts of organic acids, such as tartrates, citrates, malates, &c., abound in the food of herbivora, while in that of the carnivora and man, the phosphatic salts prevail, we have a very simple explanation of the discrepancy. In the latter, little or no change takes place in the constitution of these salts ingested in the food ; in the former, there is a transformation of the organic acids into carbonic acid, and hence the preponderance of carbonates. Verdeil, on analysing the ashes of the blood of these different classes of animals, found the same diversity of constitution. Thus in the blood-ash of the ox, he found only 3 per cent. of alkaline phosphates, while from that of a dog fed on meat, he obtained 12 per cent. of these salts. In the ash of human blood, the normal proportion of phosphates is about 10 per cent.

